# Protocol Design for Surveillance of Risk Factors of Non–communicable Diseases During the COVID-19 Pandemic: An Experience from Iran STEPS Survey 2021

**DOI:** 10.34172/aim.2022.99

**Published:** 2022-09-01

**Authors:** Shirin Djalalinia, Sina Azadnajafabad, Erfan Ghasemi, Moein Yoosefi, Negar Rezaei, Yosef Farzi, Ameneh Kazemi, Naser Ahmadi, Maryam Nasserinejad, Nima Fattahi, Shahabeddin Rezaei, Elham Abdolhamidi, Elmira Foroutan Mehr, Rosa Haghshenas, Nazila Rezaei, Jaleh Abdi, Alireza Moghisi, Alireza Mahdavihezaveh, Ali Akbari Sari, Alireza Raeisi, Hamidreza Jamshidi, Bagher Larijani, Farshad Farzadfar

**Affiliations:** ^1^Non-Communicable Diseases Research Center, Endocrinology and Metabolism Population Sciences Institute, Tehran University of Medical Sciences, Tehran, Iran; ^2^Development of Research and Technology Center, Deputy of Research and Technology, Ministry of Health and Medical Education, Tehran, Iran; ^3^Endocrinology and Metabolism Research Center, Endocrinology and Metabolism Clinical Sciences Institute, Tehran University of Medical Sciences, Tehran, Iran; ^4^Department of Biostatistics, Faculty of Paramedical Sciences, Shahid Beheshti University of Medical Sciences, Tehran, Iran; ^5^Ohio State University, Columbus, OH, USA; ^6^National Institute for Health Research, Tehran University of Medical Sciences, Tehran, Iran; ^7^Deputy of Health, Ministry of Health and Medical Education, Tehran, Iran; ^8^Department of Health Management and Economics, School of Public Health, Tehran University of Medical Sciences, Tehran, Iran; ^9^School of Medicine, Shiraz University of Medical Sciences, Shiraz, Iran; ^10^Research Institute for Endocrine Sciences, School of Medicine, Department of Pharmacology, Shahid Beheshti University of Medical Sciences, Tehran, Iran

**Keywords:** COVID-19, Iran, Non-communicable Disease, Population Surveillance, Protocol, Risk Factors, STEPS

## Abstract

**Background:**

Regarding the growing burden of non-communicable diseases (NCDs) and exposure to their risk factors, and the continuous need for nationwide data, we aimed to develop the latest round of the STEPwise Approach to NCD Risk Factor Surveillance (STEPS) survey in 2021 in Iran, while the COVID-19 pandemic was still present.

**Methods:**

In addition to the three main steps of this survey, including questionnaires, physical measurements, and laboratory assessments, we adapted the survey with the situation caused by the COVID-19 pandemic, by adding to various aspects of study phases and changing some scientific and executive procedures in this round of STEPS survey in Iran. These changes were beyond the initial novelties embedded within the survey before the pandemic, by refining the study protocol benefiting from the previous experiences of the STEPS survey.

**Results:**

By amending the required changes, we could include a total of 27874 individuals in the first step of the survey. This number was 27745 and 18119 for the second and third steps. Comparing the preliminary results with the previous nationwide surveys, this study was highly representative on both national and provincial levels. Also, implementing the COVID-19 prevention and control strategies in all stages of survey led to the least infection transmission between the study investigators and participants.

**Conclusion:**

The novel initiatives and developed strategies in this round of Iran STEPS survey provide a state-of-the-art protocol for national surveys in the presence of an overwhelming catastrophe like the COVID-19 pandemic and the triggered limitations and shortages of resources.

## Introduction

 The increasing burden of non-communicable diseases (NCDs) has become a significant health priority that requires serious attention and prompt action. NCDs account for over 74.4% of deaths worldwide, and over two-thirds of these deaths occur in developing countries.^1,2^ Among them, cardiovascular diseases (CVDs) and stroke account for most deaths in the Middle East region.^1,3,4^ The most important risk factors of NCDs in this region are nutritional risk factors, hypertension, increased body mass index, smoking, insufficient physical activity, and hyperglycemia.^5,6^ Iran, as a developing country located in this region, is experiencing a transition period, and the prevalence and burden of NCDs are growing vigorously in the country.^7-10^

 The World Health Organization (WHO) action plan on control and prevention of NCDs and Sustainable Development Goals (SDGs) (target 3.4) request member states to reduce 25% of unconditional probability of deaths due to four major NCDs including CVDs, diabetes, cancers, and chronic respiratory diseases by 2025, through a reduction in tobacco use, unhealthy diet, lack of physical activity and harmful alcohol use. Therefore, this action plan urges countries to have reliable data on the levels and distribution of NCD risk factors.^11-13^

 In Iran, considering the national NCDs action plan and specific targets for national and sub-national levels, governors and medical universities administrators need access to reliable evidence for monitoring their NCD prevention and control committees at provincial and district levels.^14^ This information helps them to monitor and fully implement the nationally-endorsed strategies.^15-17^ Considering the capacity of national surveys in Iran, the WHO STEPwise Approach to NCD Risk Factor Surveillance (STEPS) framework effectively responds to this data need.^18^ The STEPS framework is committed to collecting, analyzing, and interpreting the required information and providing them to beneficiaries according to the systematic guidelines and standards of the WHO.

 So far, seven rounds of the STEPS surveys have been implemented in Iran in 2005, 2006, 2007, 2008, 2009, 2011, and 2016. To improve guidelines and protocols and to optimize web-based processes in Iran, the protocols and instruments of the STEPS survey 2021 included three different levels of assessment: step 1) demographic, epidemiologic, and risk-related behavioral data collection; step 2) physical measurements, and step 3) lab measurements all among nationally and sub-nationally representative sample of Iranian adults. The data provided in this survey enables health policymakers to monitor and evaluate NCD action plans and additionally provides capacity for medical schools and other stakeholders to benefit from the data.^17,19-21^ The successful experience of the previous round of Iran STEPS survey 2016 by the Non-communicable Diseases Research Center (NCDRC) enabled us to design this round more effectively and constructively.^18,19^

 Compared to the previous seven rounds of STEPS survey implementation, we encountered an unpredicted crisis, the COVID-19 pandemic, during this national survey.^22^ Due to safety requirements, the sampling process stopped when we completed about 10% of the estimated sample size. We turned the pandemic threat into an opportunity to estimate the interaction between the COVID-19 and NCDs by completing a national survey while collecting the remaining 90% of samples.^23^ The process of completing the sampling continued after the third peak of the disease when the incidence of COVID-19 reached its lowest levels. For this purpose, supplementary scientific protocols and instructions for protection and safety were developed to conduct the survey in the COVID-19 pandemic situation. As another requirement, ethical and legal permits were obtained from the related authorities. The present study describes the scientific and executive features of the recent STEPS survey in Iran and the details of the standard settings and protocols before and during the COVID-19 pandemic.

## Overview

**Figure 1:**
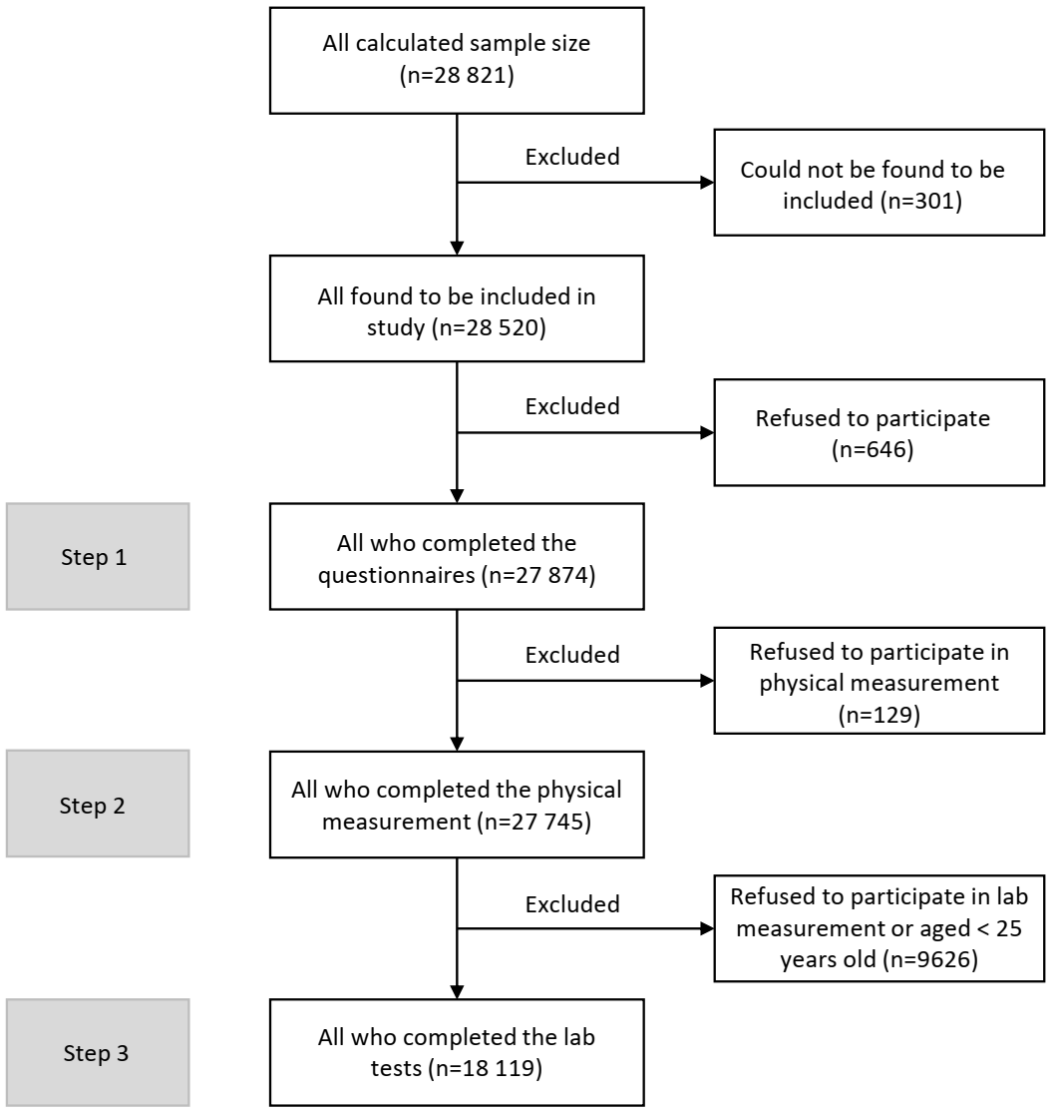
Details of the Number of Participants Included in Each Step of the Survey and the Number of Excluded Participants

 Here, we provide an overview of the study design and protocol. This cross-sectional study started in early 2020 under the supervision and management of the online project management system and was suspended due to the COVID-19 pandemic after collecting about 10% of samples. The implementation phase of the study restarted in early 2021. The primary sample size was estimated at 31 760 individuals from 3176 clusters across the country. During the survey re-implementation, based on the renewed estimations, we followed the sampling for 28 821 individuals from 3176 clusters. Among the estimated population, 28 520 adults were found to be included in the survey and among them, 27 874 individuals who provided voluntary informed consent were included in the study. The number of participants for the survey steps was 27 874 for the first step, 27 745 for the second step, and 18 119 for the third step (Figure 1).

 The measurements of this survey focused on the exposure to the main risk factors of NCDs in Iran. Following the WHO standards and considering the requirements and needs of the country, the first step was designed based on questionnaires focused on the prevalence of major metabolic, nutritional, and behavioral risk factors, in addition to some specific medical history of NCDs. The second step was composed of physical measurements (anthropometry) and the third step was designed for laboratory measurements.

 Considering the importance and interaction of NCDs and the COVID-19 infection, the study design was refined for better planning and design of more effective interventions, and a few items were added to the questions. Considering the scientific standards for collecting survey data along with maintaining the health and safety of various stakeholders, including study participants, executive groups of questioning and anthropometry, supervision, sample transfer and laboratory investigators, and other collaborators, development of protection and safety protocols of target groups was given special attention. Therefore, one of the requirements at the resuming of the survey was to instruct these protocols to the target groups. Regarding the conditions resulting from the COVID-19 pandemic and the emphasis on distance learning methods, the material and education were provided mainly in online sessions. Therefore, all study collaborators and participants in urban and rural areas participated in this survey according to standard instruction. Based on the test results of biological samples and monitoring reports, fortunately, no incidence of COVID-19 due to study implementation was recorded during the survey.

## Sampling Frame and Sample Size Estimation

 In every STEPS survey, the sample should represent the target population. Therefore, designing and selecting a standard sample to obtain valid and representative results are essential. Five critical components were considered for sampling efficiency and accuracy in this survey including the optimal confidence interval, an acceptable margin of error, design effect, estimated baseline index level (prevalence of indices), and the non-response rate. The sample size required for evaluating risk factors of NCDs in each province was calculated using a proportion to population size method. Details on each incorporated factor for sample size calculation and the final calculated number of individuals for each province are provided in Supplementary file 1.

 Due to study complexity, financial constraints, regional differences, and heterogeneous population distributions, we used the systematic cluster classification method. This method combines different probability sampling methods. A total of 3176 clusters, each with 10 participants, was calculated to conduct the study on the national level. This number was estimated based on each province’s population; therefore, the relative weighting was different for each one. After the outbreak emerged in Iran, to complete the remaining 90% of sampling, due to financial constraints and COVID-19 precautions and limitations, one person from each cluster was reduced and the clusters were completed with 9 participants (due to necessary considerations during the analysis of the sample weight). Through this sampling method, we designed a systematic cluster random sampling frame through which 28,821 participants from 3176 clusters were selected from rural and urban areas of the 31 provinces of Iran.

## Inclusion and Exclusion Criteria

 Based on the eligibility and sampling criteria of the 2021 STEPS survey, the Iranian adults aged above 18 who resided in urban or rural areas of one of the 31 provinces of Iran were studied as the target population. For the included population, demographic information and metabolic and behavioral risk factors were evaluated using questionnaires, anthropometry, and laboratory (only for participants aged ≥ 25 years). The following individuals were excluded from the study: people with mental disorders who may be unable to answer the questionnaires, people for whom anthropometry measurement was impossible due to physical limitations, people who could not provide laboratory samples, and pregnant women. Data were collected from individuals who agreed to participate and provided informed consent.

## Questionnaires and Interview Protocol (Step One)

 As one of the noteworthy features in developing the STEPS questionnaire, the primary basis was the standard questionnaire developed by WHO. The latest version of the WHO questionnaire (version 3.2) was used for this purpose.^24^ The STEPS questionnaire consists of two sections of core and expanded questions. This survey’s questionnaire included all core questions and most of the expanded questions including expanded demographic, diet, physical activity, and tobacco use. In addition, some questions were added to the questionnaires list due to national concerns and policies for prevention and control of NCDs, named as modified and newly added questions here, including modified diet (the most important were the food labeling and fish consumption), physical activity, tobacco use, alcohol consumption, injuries with a particular emphasis on road traffic injuries, cancer screenings (prostate cancer for men, cervical and breast cancer for women, colorectal cancer for both), and history of human papillomavirus (HPV) vaccine immunization for women.

 In order to design and validate this round of STEPS questionnaires in Iran, a team of experts in the fields of population health sciences, medicine, epidemiology, health education, nutrition, geriatric, and oral health specialists was formed. Details of various parts of the questionnaire, physical measurements, and lab tests in the three steps of this study are provided in Table 1.

**Table 1:** Details of Various Sections of the Questionnaires, Physical Measurements, and Laboratory Tests in the Three Steps of the Iran STEPS Survey 2021

**Step of Study**	**Recorded Data**	**Question/Measure/Test**	**Details**
First	Questionnaires	Demographic information	Location, ID, age, sex, marital status, education, job status, insurance (basic, complementary)
		Diet	Number of meals, snacks, breakfast, fruits, vegetables, dairy (low-fat, semi-fat, high-fat), meat, fish, processed meat, rice, bread, whole grains, sweet beverages, nuts, food labeling, nutritional facts, salt-shaker use, salt use, salty processed foods, attitude toward salt use reduction
		Physical activity	Vigorous-intensity activity, moderate-intensity activity, walking, biking, vigorous-intensity sports, moderate-intensity sports, sedentary behavior, obstacles of physical activity
		Past medical histories	Hypertension (awareness, treatment, type of medication/herbal medication), Diabetes mellitus (awareness, treatment, type of medication/herbal medication/Insulin/Pen insulin), Hypercholesterolemia (awareness, treatment, type of medication/herbal medication), cardiovascular diseases (ischemic heart disease, angina pectoris, stroke, statin and aspirin use, interventional treatment), cancer, asthma and chronic obstructive pulmonary disease, menopause (females), dental pain, edentulism, oral health
		Lifestyle advice	Tobacco use cessation, salt use reduction, fruit and vegetable consumption, fat use reduction, processed food use reduction, fish use, whole grains use, sweet beverages use reduction, physical activity, weight control
		HRQoL	Mobility, self-care, usual activities, pain/discomfort, and anxiety/depression
		Cancer screening	Prostate cancer for men, cervical and breast cancer for women, colorectal cancer for both, Human papillomavirus (HPV) vaccine injection for women
		Injuries	Type of injury, time of injury, injury healing, seatbelt (front and back/driver and non-driver), and helmet use
		Tobacco use	Ever and current tobacco, cigarette, hookah, pipe, electronic cigarette, and smokeless tobacco use, onset age, try to stop using, advice to quit, second-hand smoke
		Alcohol consumption	Type and amount of alcohol, binge drinking, driving being drunk, or being in a car with a drunk driver
		Family asset	House, car, furniture, other facilities
Second	Physical measurements	Height	With a plausible range of 100-270 cm
		Weight	With a plausible range of 20-350 kg
		Waist circumference	With a plausible range of 30-200 cm
		Hip circumference	With a plausible range of 45-300 cm
		Blood pressure	Systolic blood pressure with a plausible range of 40-300 mm Hg Diastolic blood pressure with a plausible range of 30-200 mm Hg
		Pulse rate	With a plausible range of 30-200 beats per minute
		Pedometry	In a subsample of study, with a maximum plausible of 50 000 steps per 24 hours
Third	Laboratory measurements	Anti-SARS-CoV-2 IgG test	—
		Total serum cholesterol	—
		Serum HDL-C	—
		Serum TG	—
		Serum creatinine	—
		Urine creatinine	—
		Serum BUN	—
		Serum ALT	—
		Whole blood HbA1c	—
		Fasting plasma glucose	—
		Urine sodium	—
		Urine potassium	—
		Urine albumin	—
		Urine creatinine	—
		24-Hour urine sodium and creatinine	for the sub-sample of 609 participants randomly selected

HRQoL, Health-related quality of life; HDL-C, high-density lipoprotein cholesterol; TG, triglyceride; BUN, blood urea nitrogen; ALT, alanine transaminase.

 Health-related quality of life (HRQoL) assessment was one of the parts added to this survey that was assessed by the EuroQol five-dimensional at three levels (EQ-5D-3L) questionnaire gathering data on five fields of mobility, self-care, usual activities, pain/discomfort, and anxiety/depression with answers in three levels of no problem, slightly having a problem, and debilitated by the condition. Besides, a global scoring between 0 and 100 to the overall self-perceived health status was recorded.^25^

 The face validity and content validity of the questionnaires were assessed. In this regard, 35 individuals from all ages with different levels of education and social status were included for validation. The original questionnaire underwent two phases of forward and backward translations to ensure its validity. Through the qualitative phase, the wording of the questions was modified based on the interviewees’ feedback and expert opinion. Content validity index (CVI) and content validity ratio (CVR) were used to assess content validity. To assess the relevance of the questions, the final questionnaires were provided to 17 experts to feedback on the relevance, transparency, clarity, simplicity, and necessity, according to the checklist.

 The questioning guideline was developed and provided for the interviewers. Protecting all individuals’ privacy in data collection was a principal rule in this survey. Each interview team consisted of male and female interviewers to match individuals and facilitate their cooperation regarding gender issues. More private subjects, for example, questions about tobacco use and alcohol consumption, were asked privately to record the most accurate and honest data.

 One of the beneficial outputs of the interview step was calculating the wealth index (WI) based on the factors assessed via the household assets questionnaires which asked about various dimensions of the participants’ assets. In this regard, 36 questions examining the related aspects were included and the principal component analysis (PCA) method was utilized to summarize the multi-variable gathered data. Using the PCA method enabled us to produce new main descriptive variables known as the principal components encompassing all recruited primary variables. The first component, including most of the variables’ data, was assigned as the WI and categorized into five quintiles; the first was representative of the poorest and the fifth was representative of the wealthiest quintiles of the included population.^26^

 Strict COVID-19 specific protections were implemented for the period of the survey that happened during the pandemic. Before starting the interview, any suspicion of COVID-19 symptoms or an approved diagnosis of disease within the past two weeks in the individuals or interviewers was the contraindication of the interview. In case of the absence of these criteria, interviewers started to record the data and conduct physical measurements with special considerations like using face shields, masks, and gloves. COVID-19 related items were also embedded in the data collection process for all individuals after the beginning of the pandemic to ask about the COVID-19 symptoms and diagnosis in the individual, family, and other people within their contact.

 In terms of implementation protocol, ruling out the COVID-19 infection in the interviewer through checking the signs and symptoms recorded as beginning questions of the software was done every day. Safety protocols through interviews included maintaining the necessary social distancing, wearing masks, and disinfection of hands and measurement devices. Also, participants were asked about the COVID-19 symptoms, access to and use of outpatient services, disease outcomes, compliance with health protocols, access to online health services, and family behaviors regarding the pandemic.

## Physical Measurements Protocol (Step Two)

 The physical measurement step was designed to assess the participant’s height, weight, waist circumference, hip circumference, blood pressure, and pulse rate. Following the standard protocol, for all participants aged ≥ 18 years, the measurements followed the WHO criteria. Wearing masks by both the interviewer and participant and making the measurements in the shortest time and with a safe distance was mandatory during the measurements to prevent COVID-19 transmission. Also, the COVID-19 prevention protocol was developed and instructed to interviewers to maximize the safety considerations. All measurements were made by matched gender examiners and in a place where participants were comfortable. Data were gathered from 27 745 participants. Details of physical measurements are available in Table 1.

 Height was measured by a standard meter in standing position straight against a wall, with heels, hip, and back of the head in a straight line relying on the wall. Weight was measured with a standard digital scale (Inofit) and calibrated with an index scale of 5 kg before use each time the device was moved. Waist circumference was measured halfway between the lowest rib and hip, almost crossing the umbilicus, in a straight line. Hip circumference was measured on the broadest round of the hip. Pulse rate was measured in a sitting position after 60 seconds. Systolic and diastolic blood pressures were measured in three rounds on the brachial artery, each with a three-minute interval, starting after 15 minutes of rest in a sitting position, by standard Beurer sphygmomanometers. The mean of the second and third measurements was reported as the final blood pressure value. The mentioned measurements were made in all included eligible participants in the second step. A further examination of pedometry in the last 24 hours (by Xiaomi pedometers) was conducted in a subsample of participants to actively measure their physical activity through their step counts and burnt calories. The measurement instruments were calibrated in the preparation phase of the survey, and only instruments that successfully passed the standardization process were packaged and delivered to the local investigators.

## Laboratory Measurements (Step Three)

**Figure 2:**
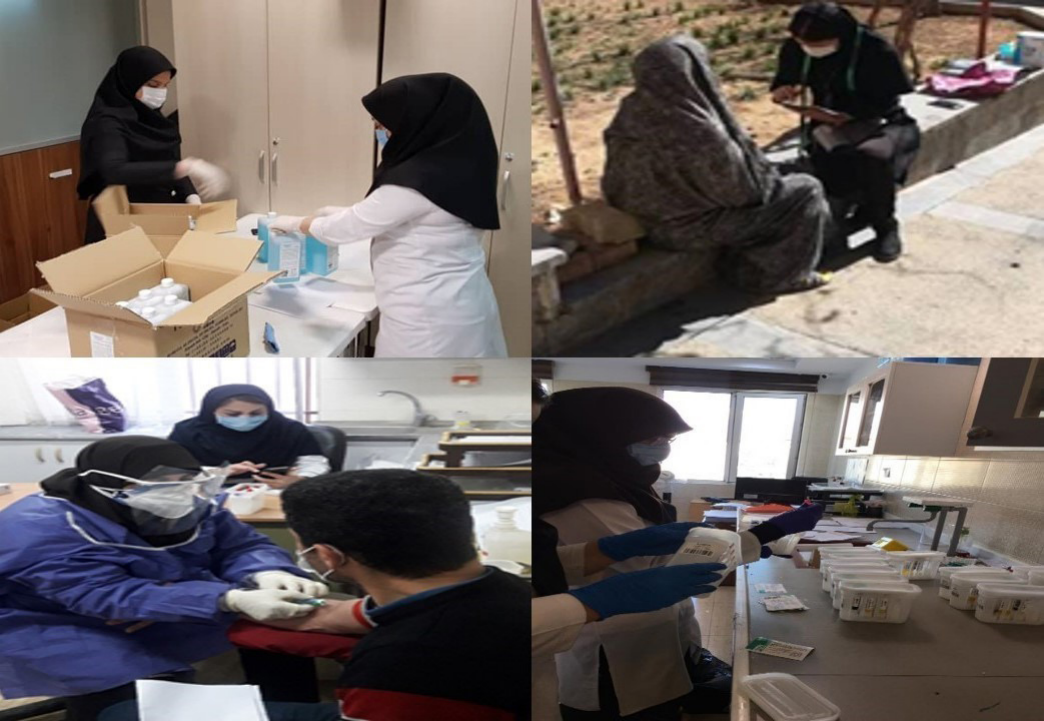
Study Considerations During the COVID-19 Pandemic

 The target population for the third step was individuals at least 25 years of age of both sexes. This step was intended to identify several risk factors of NCDs through biochemical blood and urine tests. The third step happened in seven stages including recruiting participants and allocating specific barcodes to each individual, obtaining samples, primary processing of samples by centrifuging and aliquoting, temporary storage of samples, transportation of samples, performing tests on the samples at the central lab of study, and finally biobanking the samples for further investigations in future. In order to perform the third step of the study safely, for all stages of acceptance, sampling, isolation, storage, and transporting samples, instructions were set based on the standards of personal protection against COVID-19 (Figure 2).

**Figure 3:**
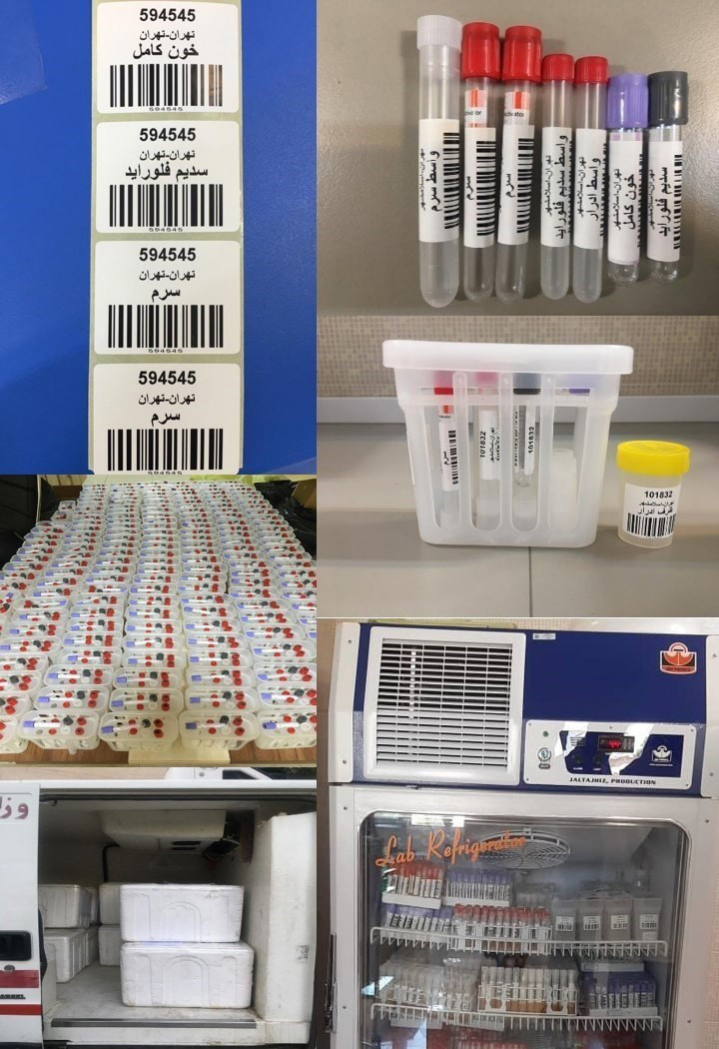
Study Equipment and Stages of Sample Obtaining, Storing, Transferring, and Analyses

 Maintaining the optimal conditions for the transfer of biological samples using the updated standards for promotion of quality of biological samples and biomolecules maintenance, we developed the comprehensive participatory protocol and related instructions. After blood and urine sample collection, the transport was performed at a temperature of 4ºC. Using a specifically designed package including five tubes containing whole blood (5 cc), buffy coat (1 cc), plasma obtained from the tube containing lithium heparin (3 cc), and urine (6 cc), the gathered biological samples were transferred to the central processing/archiving laboratory of the study at NCDRC. Through a detailed time-binding action plan, the processes from collection to the central lab were managed in the shortest time (less than 18 hours). All samples were transported in vaccine transport boxes. For all of the stages requiring storing and transferring of the samples, the cold chain (2-8ºC) was provided to ensure the quality of samples. During transportation, in each cold box, a digital thermometer recorded the temperature of the environment of the biological samples. These enabled us to keep the samples from freezing/thawing (Figure 3).

 Using the auto-analyzer (Roche-Hitachi Cobas C311, High–Technologies Corporation, Tokyo, Japan), which the reference laboratory approved, the lab tests of serum total cholesterol, high-density lipoprotein cholesterol (HDL-C), triglyceride, creatinine, alanine transaminase (ALT), blood urea nitrogen (BUN), whole blood HbA1c, fasting plasma glucose, and urine sodium, potassium, and creatinine were performed. Urine albumin was assessed by Prestige premium 24i analyzer. To evaluate the validity of spot urine samples, 24-hour urine sodium and creatinine assessments were done for a subsample of participants randomly selected from all over the country. For the study phase during the COVID-19 pandemic, an additional serum test of specific anti-SARS-CoV-2 IgG was assessed by BioTek ELISA reader (Table 1). After conducting the biochemical tests with the auto-analyzer, the test results were automatically sent to the server. The device operator checked the test results using the laboratory process management software (LabIt) and repeated the tests if needed.

## Data Management System

 To accelerate and facilitate the implementation of the processes and to improve the accuracy of data registration, the information technology (IT) standards and guidelines were utilized. Regarding the questionnaire design for tablets, a software under the Android operating system was designed and finalized after testing the qualitative content of the data collection questions considering the sampling conditions mentioned in the relevant sections. The final version was installed on all tablets for interviewers and was updated continuously while training the interview teams. The information collected through this software was generally divided into the following categories: information related to the questionnaires, anthropometry, laboratory assessment, information related to different levels of supervision, and survey quality control. This information was stored on the tablets and transported for final storage on the study server via an online internet connection.

 As one of the main steps of the survey, the LabIt software’s main tasks included identifying obtained samples, locating and managing them, and generating system reports so that the sample’s condition and identity may be known from the sample’s admission until their placement in the auto-analyzer. The software’s processes were designed according to the high error coefficient of sampling and allocation to minimize errors. The main survey software connected the research server to the Cobas C311 auto-analyzer and transferred test results from the device to the database.

## Capacity Building and Training

 Although at the beginning of the study training and empowerment workshops were conducted separately for the target groups at different levels of implementation, in the phase of completing the study, following the initial advocacy and necessary coordination, supporting workshops were held to update the information of more than 700 audiences from 62 universities and medical schools, over five days. We also defined and developed a comprehensive package of hierarchical training steps for different levels of study partners. Developed documents contained curricula and educational materials which were prepared based on predefined involvement roles and expectations of different partners. Aimed at better accessibility, all packages were distributed through the study website, as a Training of Trainers (ToT) model. Educational methods are essential due to their impact, the variety of subjects, and the survey’s multiple cooperative administrative levels. Therefore, an effort was made toward other methods, including tutorial preparation, providing essential hints using the questionnaire software (simultaneous access during the interview), access to related training videos, as well as continuous training through constant communication with the target groups that were included in plans in addition to designing and implementing educational workshops.

 Another noteworthy point was the need for participating in online exams and obtaining a passing score to enter the study as an administrative colleague or a member of the supervisory network. The details of training and empowering the individuals selected and introduced for participation in the general survey and specialized courses were inserted in specifically related protocols.

## Data Cleaning and Analysis

 Similar to the previous round, the data for the STEPS survey 2021 was collected via tablets. It was essential to use tablets and make the questionnaires electronic since it could solve most of the problems associated with paper questionnaires, including missing data, unacceptable data, etc. This section concerns data extraction from a central database for data cleaning, weighting, description, and analyses for the three steps of the survey.

 The data cleaning process starts with differentiation between questions with missing answers and questions without answers due to jumps in the questionnaire. The first step was identifying the implausible range of variables and outlier values. Any data out of the plausible range was considered an outlier. Questions with answers of “I do not know” were considered missing data. For higher quality data cleaning, the process was conducted separately by two biostatisticians, and a third data expert resolved the discrepancies. Also, for more accurate data cleaning, the process was done in both R and STATA software, and the results were compared. The second step identified the missing data. In case of a missing answer for the key question of each section of the questionnaire (usually the first question in each section), the whole section was assumed as missing data. Missing data identification was conducted by two biostatisticians, as well. The third step was properly detecting jumps and allocating suitable values for each jump in case of a negative answer to that question.

 A major step after data cleaning and before data analysis was data weighting since it was needed to address the non-response and incomplete data collection added to different characteristics of populations included in this survey in order to make the results valid and reliable. In this regard, a weighting procedure was deployed to adjust the survey results based on various factors. In brief, the weighting procedure for the cleaned data in this survey was done in four stages including (a) weighting for general non-response defined as not participating in the study at all, (b) weighting for non-response at each step of questionnaires, anthropometry, and laboratory measurements, (c) weighting for age, sex, and area of residence, and (d) the final weighting for the analysis of data of each step of the survey based on the three calculated weightings. The details on the weighting steps and the equations are provided in Supplementary file 2.

## Quality Control and Supervision

 Quality control and supervision happened in different steps and stages of the study to ensure the quality of the process and survey results. For instance, during step one, the interview with participants was recorded for secondary evaluation by managers at different levels. Also, selected parts of the interviews were randomly chosen and the questionnaires were filled with the recorded voices and the results were compared to the information recorded in-person on the system. Supervision and monitoring checklists were another tool of quality control completed at local levels by investigators and were available online through the survey data management system. For steps two and three, all instruments and analyzers were calibrated and checked to receive the highest standards prior to utilization in different stages. Also, an additional round of quality checks was conducted after data collection between the data gathered before the COVID-19 pandemic and the data collected during the pandemic. This investigation showed that the results had no statistical difference and merging all data into one unique dataset was possible.

 Considering the broad scope of the STEPS survey and its simultaneous execution across the country, a system was deployed for supervising the survey’s various processes as well as producing different reports. In this regard, a central management panel was developed to simultaneously supervise the data gathering process via the online data system at the survey headquarter at NCDRC. Supervision started prior to allocating clusters to interviewers. It included online tests, online training, deployment control, administrative control, laboratory control, various national, university, and urban supervision checklists, supervising and controlling sampling advancement and data quality, controlling interviewer deviation, and monitoring interviewer error according to the questions. Overall project progress and equipment management was another task of the supervision process.

## Initiatives

 As a matter of the COVID-19 pandemic, the survey implementation with special considerations may be the most outstanding achievement of this round of STEPS survey in Iran. Due to maintaining safe conditions during the pandemic, stopping the study until the appropriate time to complete the study in addition to the need to review protocols and prepare protective requirements, were the significant challenges. We tried to turn this obstacle into an opportunity to redesign and resume the survey to access comprehensive national and sub-national representative data on the interaction of NCDs’ risk factors and COVID-19. Here, we present some of the specifications of this experience.

## Reports

 Considering the predefined outputs, STEPS survey 2021 led to the production of reliable data and reports of the national and sub-national survey on risk factors of NCDs. There will also be many fact sheets designed based on specific target groups. The VIZIT health information interactive visualization system is one of the achievements of this survey that provides the possibility of timely access to the information required by researchers, executives, and policymakers on credible evidence. Additionally, comprehensive courses on management, prevention, and control of NCDs are other practical outcomes of the project designed and implemented based on the identified needs and health priorities in each of the specialized and geographical areas for courses participants.

## Participation Rates

**Figure 4:**
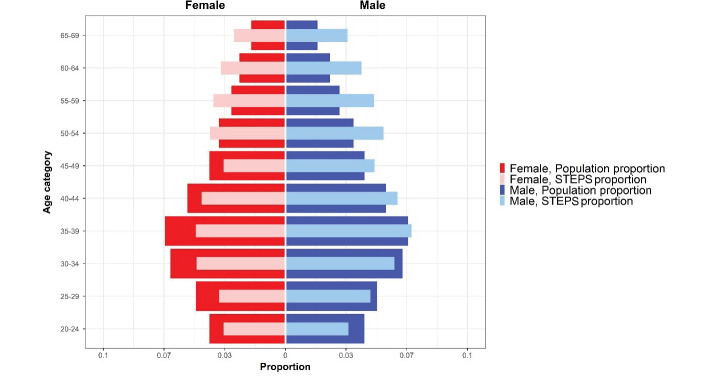
Population Pyramid of Iran STEPS Survey 2021 Sample and Iran’s Population Proportion

**Figure 5:**
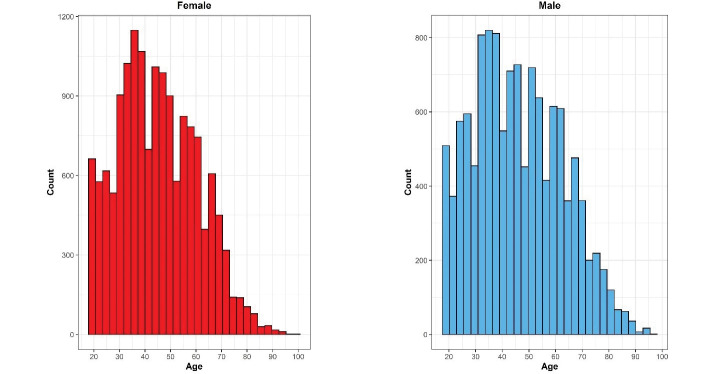
Distribution of the Included Sample in Iran STEPS Survey 2021, Stratified by Sex

 Despite the severe constraints due to the COVID-19 pandemic, the cooperation of all partners on the national and district levels, and the incentives to attract the cooperation of participants, the survey addressed the predefined response rates for all three steps of the survey. A comprehensive chart of participation in different survey steps is presented in Figures 1, 4, and 5.

## Representativeness

**Figure 6:**
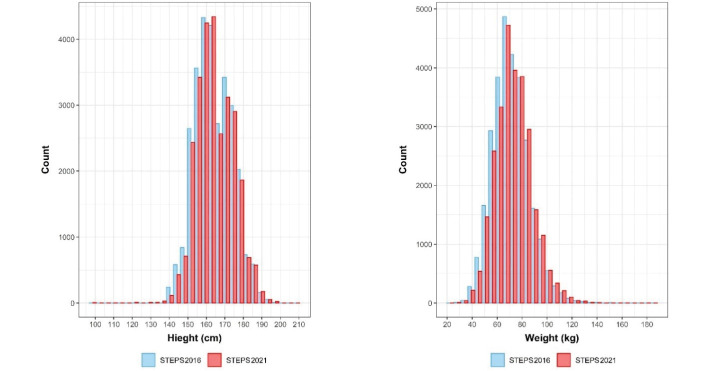
Distribution of Height and Weight of Participants in Iran STEPS Surveys 2016 and 2021

 Based on applying sampling weight on the gathered samples, we assessed the distribution of population and samples using the Kolmogorov–Smirnov compared classification according to the age and sex groups. Probability sample tests showed that the study population was representative on the national and provincial levels. A comparison was made between the included sample in the STEPS survey 2021 and the estimated age and sex combination of Iran’s total population provided by the Statistical Center of Iran, and the differences in this comparison were not statistically significant (*P*-value = 0.675) (Figure 4). Also, the histogram of the STEPS 2021 sample had normal distribution for both sexes (Figure 5). Another comparison between the height and weight distribution of individuals in STEPS 2021 and the previous STEPS survey 2016 in Iran, whose representativeness had been proven before, was made, and the distribution of samples was almost the same in both samples (Figure 6).

**Figure 7:**
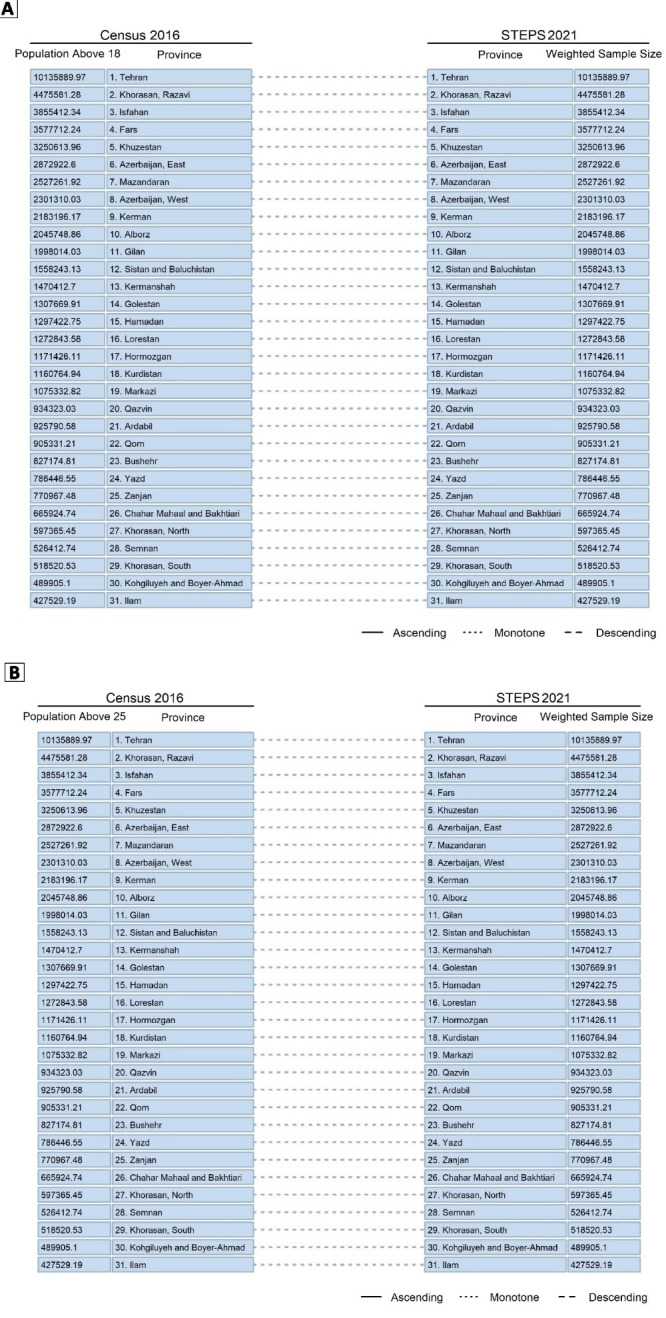
Comparison of the Rank of the Population of Provinces and the Adjusted Sample Size of Provinces in STEPS Survey 2021, (A) for the Population Above 18 Years, and (B) for the Population Above 25 Years of Age

 An additional analysis of the recruited weights of sample for each province based on the resident population of provinces and comparing the results with Iran’s latest census in 2016, in two analyses of the population above 18 years and the population above 25 years of age, showed that after adjusting the samples with the relative weights, the rank of the population was accordingly the same as the actual population of provinces. This finding was another proof of the appropriate sampling and clustering methods used in this study and the national representativeness of the survey (Figure 7).

## Electronic Health Certificate

 Electronic health certificate was another initiative that helped enhance study participation, provided timely and online access to biological test results and anthropometric information along with relevant interpretations and suggestions for follow-up and health promotion for participants.

 Benefiting from an artificial intelligence system, integrated results from the questionnaires, anthropometric and laboratory steps will be provided to each participant in an electronic health certificate. In addition to summarizing the results, this report provides the risk score for CVDs and diabetes. The link to this electronic record will be sent automatically to participants through texts to allow them to view their record by visiting the SEPID platform. For the participants without internet access, reports will be printed out and mailed.

## Chronic Kidney Disease

 Due to the considerable burden and high cost of prevention and management of chronic kidney disease (CKD) in Iran, for the first time, we planned to estimate the prevalence of CKD based on the calculation of estimated glomerular filtration rate (eGFR) using patients’ age, sex, and the measured serum creatinine, in addition to the measured urine albumin/creatinine ratio for all the participants recruited in the third step of survey. We hope this evidence will help planners and policymakers to design and implement more effective interventions.

## Food Product Labeling

 As a public health intervention to improve the dietary intake of consumers, knowledge on food product labeling known as the “traffic light labels” was assessed for the first time through this survey. Awareness of the importance of the food product labels and their role in participants’ choice of the products were assessed by newly developed questions embedded in the first step of this survey.

## Cancer Screening

 As an opportunity for comprehensive cancer surveillance as a significant component of NCDs, screening for common cancers was assessed first in this national survey. The history of cancer screening was assessed for colorectal cancers (positive occult blood test and colonoscopy reports), prostate cancer (positive prostate-specific antigen (PSA) blood test) for men, cervical cancer (positive Pap smear and HPV test), and breast cancer (positive mammography reports) for women.

## COVID-19 IgG Antibody Test

 Due to the outbreak of COVID-19, for the completion phase of this survey in 2021, the COVID-19 IgG antibody evaluation was added to this survey. One of the innovations of this study was tracking the people who tested positive. According to the data registry based on tracking results, prompt warnings were reflected to the at-risk family members. Moreover, the study executive teams in contact with the infected person were followed up.

## Other Initiatives

 This survey also had some other novelties in the questionnaire step, including gathering data on the history of premature menopause in women, detection of familial susceptibility to CVDs, attention to labels and warning signs on cigarette packs, binge drinking, driving under inebriation or being in a car with a drunk driver, etc. These assessments will be reflected in further reports and papers to provide a clearer picture of the latest status of the NCD risk factors in Iran.

 In conclusion, to the best of our knowledge, this is the first national STEPS survey conducted during the COVID-19 pandemic based on standard protection and safety protocols, globally. Despite the limitations and challenges of implementing this survey during the COVID-19 crisis, some initiatives in different study steps made this round of STEPS survey unique compared to the previous rounds. Benefitting from the cooperation of all the scientific and executive stakeholders of the health section, we were able to reach the highest possible level of participation and standards in this survey. The results of this study would enable policymakers, health authorities, and researchers to more accurately estimate the risk factors of NCDs in Iran for the final goal of designing and implementing more effective intervention programs regarding NCD prevention and control.

## Supplementary files


Supplementary file 1. The details of sample size calculation for Iran STEPS survey 2021.

Supplementary file 2. The details of the weighting procedure of the cleaned data of Iran STEPS survey 2021.
