# Supplementary material for: Protocol Design for Surveillance of Risk Factors of Non–communicable Diseases During the COVID-19 Pandemic: An Experience from Iran STEPS Survey 2021
Source: Arch Iran Med. 2022 Sep 1;25(9):634–46. doi: 10.34172/aim.2022.99 (PMC10685773; doi:10.34172/aim.2022.99)
Supplement: Supplementary file 1 — The details of sample size calculation for Iran STEPS survey 2021. [file aim-25-634-s001.pdf]

Arch Iran Med. September 2022;25(9):634-646
10.34172/aim.2022.99

Protocol Design for Surveillance of Risk Factors of Non–communicable Diseases During the COVID-19 Pandemic: An Experience from Iran STEPS Survey 2021

Shirin Djalalinia, PhD[1,2]; Sina Azadnajafabad, MD, MPH[1]; Erfan Ghasemi, PhD[1]; Moein Yoosefi, MSc[1]; Negar Rezaei, MD, PhD[1,3]; Yosef Farzi, MSc[1]; Ameneh Kazemi, MSc[1]; Naser Ahmadi, MSc[1]; Maryam Nasserinejad, MSc[1,4]; Nima Fattahi, MD[1]; Shahabeddin Rezaei, MSc[1,5]; Elham Abdolhamidi, MSc[1]; Elmira Foroutan Mehr, MSc[1]; Rosa Haghshenas, BSc[1]; Nazila Rezaei, MD[1]; Jaleh Abdi, MD, PhD[6]; Alireza Moghisi, MD, MPH, PhD[7]; Alireza Mahdavihezaveh, MD, MPH[7]; Ali Akbari Sari, MD, MPH, PhD[6,8]; Alireza Raeisi, MD[7,9]; Hamidreza Jamshidi, PharmD, PhD[10]; Bagher Larijani, MD[3]; Farshad Farzadfar, MD, MPH, MHS, DSc[1,3*]

[1]Non-Communicable Diseases Research Center, Endocrinology and Metabolism Population Sciences Institute, Tehran University of Medical Sciences, Tehran, Iran
[2]Development of Research and Technology Center, Deputy of Research and Technology, Ministry of Health and Medical Education, Tehran, Iran
[3]Endocrinology and Metabolism Research Center, Endocrinology and Metabolism Clinical Sciences Institute, Tehran University of Medical Sciences, Tehran, Iran
[4]Department of Biostatistics, Faculty of Paramedical Sciences, Shahid Beheshti University of Medical Sciences, Tehran, Iran
[5]Ohio State University, Columbus, OH, USA
[6]National Institute for Health Research, Tehran University of Medical Sciences, Tehran, Iran
[7]Deputy of Health, Ministry of Health and Medical Education, Tehran, Iran
[8]Department of Health Management and Economics, School of Public Health, Tehran University of Medical Sciences, Tehran, Iran
[9]School of Medicine, Shiraz University of Medical Sciences, Shiraz, Iran
[10]Research Institute for Endocrine Sciences, School of Medicine, Department of Pharmacology, Shahid Beheshti University of Medical Sciences, Tehran, Iran

Supplementary File 1. The details of sample size calculation for Iran STEPS survey 2021.

For a precise and effective sample size calculation, five components of the optimal confidence interval, acceptable margin of error, design effect, estimated baseline index level (prevalence of indices), and the non-response rate were deployed in the sampling method of this survey. The required sample size for each province in this survey was calculated by the proportion to size method using the population of each province. In the first stage, the sample size was calculated using the Cochran formula (#1) as follows:

$$(\#1)\ n = z_{1-\alpha}^{2}\frac{p(1-p)}{e^{2}}$$

Where the value of *z* is calculated according to the *1-α* level of confidence from the normal distribution. Also, *p* is the prevalence of the assessed variables and *e* is the margin of error. Therefore, to use this formula for sample size calculation, we need to determine the baseline factors mentioned below.

1. Confidence interval:

This factor is representative of uncertainty in the estimation of the mean/prevalence of the assessed variable in population determined by the mean/prevalence of the variable in a sample of that population. Considering 0.95 as the confidence interval for this survey, the *z* value of 1.96 was utilized for sample size calculation.

2. Prevalence:

The mean/prevalence estimates of a well-designed sample are representative of the population. Generally, the value of prevalence (*p*) is chosen based on previous similar studies. In this regard, we utilized the prevalence of primary variables of the Iran STEPS survey 2016 to calculate the sample size for the current survey. Considering various variables, we chose only one variable as the index prevalence. Higher values of *p* and *1-p* of the variable result in higher estimated variance and assuming the other parameters constant, the sample size would be greater. Considering the higher variance of the "awareness of hypercholesterolemia" index in the previous Iran STEPS survey, its prevalence was chosen as the final prevalence in the sample size formula.

3. Margin of error:

The expected margin of error is defined as half of the determined confidence interval. Smaller values of margin of error will result in greater calculated sample sizes. According to the World Health Organization (WHO) STEPS framework, the margin of error for indices with prevalence ≥10% is 0.05 and for those with prevalence <10% is 0.02.[1] Considering the later margin of error for less prevalent indices, the sample size for the current survey would be too great making the conduction of survey impossible. Based on the literature, an alternative for this problem is considering the half of prevalence for estimation of margin of error is suggested for surveillance studies with big sample sizes.[2,3] Using this alternative, the largest sample size was calculated for the "awareness of hypercholesterolemia" index. To check the validity of the final calculated sample size, we assessed its generalization to different age groups, sex stratification, age-sex categories, area of residence (urban/rural), and based on provinces and the margin of error was calculated for all categories and surprisingly the calculated values were all less than 0.05 for the mentioned assessments.

4. Design effect:

Considering the overestimation of the calculated sample's variance compared to the random sampling's variance in complex samplings like the current survey, the design effect should be considered after sample size calculation to reach more accurate estimations of indices in the chosen sample. One of the components in determining the design effect is Intraclass Correlation Coefficient (ICC),[4,5] which is defined as follows (#2):

$$(\#2)\ DEFF = 1 + \rho\ (n - 1)$$

Where ρ is ICC and n is the number of samples in each cluster. For the current study, the number of samples was 10 in each cluster. To ensure the chosen number, the ICC was calculated for various indices and promising results were achieved for each cluster regarding all indices. Considering ICC=0.05 for the "awareness of hypercholesterolemia" index, about 90% of indices achieved ICCs lower than this value, which was acceptable. Therefore, a design effect of 1.45 was determined for the "awareness of hypercholesterolemia" index and this number was multiplied into the primary calculated sample size.

5. Non-response rate:

To control the non-response bias in the sample size calculation, 10% of the calculated size was added to the primary estimated number.

Summary of sample size calculation:

Including the values of the five components in formula (#1), the final sample size was 620 in 62 clusters (each with 10 samples). This sample size was assigned to the Ilam province which had the smallest population size in the country and the sample size for other provinces was calculated according to the proportional size of their population. Besides, a final correction of the calculated sample size was done due to some executive limitations, as the financial ones were the most important. In this regard, for provinces whose the calculated sample size was >2-times and <4-times of the calculated sample size of the Ilam province as the reference province, the sample size for those provinces was divided to 2. Also, for provinces whose calculated sample size was >4-times of the Ilam province, the calculated size was divided to 4.[5,6] The final number of clusters and calculated sample sizes of rural and urban areas of all provinces are provided in Table 1. We should mention that all these calculations were made before the implementation phase of the study and the COVID-19 outbreak in Iran. Further changes in the number of samples in each cluster (9 instead of 10) happened for the phase of the study during the pandemic in Iran, as the details are available in the main text of the manuscript.

Table 1. Number of clusters and calculated sample size for each province calculated based on their proportional population size.

Province	Weight of sample (based on population)	Rural sample size	Rural clusters	Urban sample size	Urban clusters	Total number of clusters
Alborz	4	52	6	689	69	75
Ardebil	2	205	21	452	46	67
Bushehr	1	331	34	851	86	120
Chahar mahall and Bakhtiari	1	320	33	634	64	97
East Azarbaijan	4	274	28	752	76	104
Esfahan	4	156	16	1216	122	138
Fars	4	364	37	913	92	129
Gilan	4	257	26	454	46	72
Golestan	2	411	42	515	52	94
Hamadan	2	321	33	588	59	92
Hormozgan	2	367	37	484	49	86
Ilam	1	189	19	422	43	62
Kerman	4	313	32	469	47	79
Kermanshah	2	246	25	793	80	105
Khuzestan	4	256	26	898	90	116
Kohgiluyeh and Buyer Ahmad	1	307	31	400	41	72
Kordestan	2	237	24	600	61	85
Lorestan	2	306	31	590	60	91
Markazi	2	175	18	583	59	77

Mazandaran	4	377	38	532	54	92
North Khorasan	1	352	36	484	49	85
Qazvin	2	167	17	501	51	68
Qom	2	33	4	624	63	67
Razavi Khorasan	4	407	41	1185	119	160
Semnan	1	150	16	614	62	78
Sistan and Baluchestan	2	557	56	566	57	113
South Khorasan	1	300	31	438	44	75
Tehran	4	201	21	3451	346	367
West Azarbaijan	4	272	28	550	56	84
Yazd	1	169	17	970	98	115
Zanjan	1	346	35	751	76	111
Total	-	8418	859	22969	2317	3176

References

1. World Health Organization. STEPs Manual. Geneva: WHO; 2014.
2. Arya R, Antonisamy B, Kumar S. Sample size estimation in prevalence studies. Indian J Pediatr. 2012;79(11):1482-8. doi: 10.1007/s12098-012-0763-3.
3. Naing L, Winn T, Nordin R. Pratical Issues in Calculating the Sample Size for Prevalence Studies. Archives of Orofacial Sciences. 2006;1.
4. Bartko JJ. The intraclass correlation coefficient as a measure of reliability. Psychol Rep. 1966;19(1):3-11. doi: 10.2466/pr0.1966.19.1.3.
5. Tucker HG. Mathematical Methods in Sample Surveys: WORLD SCIENTIFIC; 1998. 216 p.
6. Jowell R. Measuring Attitudes Cross-Nationally. 2007 2019/12/24. London: SAGE Publications, Ltd. Available from: https://methods.sagepub.com/book/measuring-attitudes-cross-nationally.
