# Supplementary material for: Protocol Design for Surveillance of Risk Factors of Non–communicable Diseases During the COVID-19 Pandemic: An Experience from Iran STEPS Survey 2021
Source: Arch Iran Med. 2022 Sep 1;25(9):634–46. doi: 10.34172/aim.2022.99 (PMC10685773; doi:10.34172/aim.2022.99)
Supplement: Supplementary file 2 — The details of the weighting procedure of the cleaned data of Iran STEPS survey 2021. [file aim-25-634-s002.pdf]

Arch Iran Med. September 2022;25(9):634-646
10.34172/aim.2022.99

Protocol Design for Surveillance of Risk Factors of Non–communicable Diseases During the COVID-19 Pandemic: An Experience from Iran STEPS Survey 2021

Shirin Djalalinia, PhD[1,2]; Sina Azadnajafabad, MD, MPH[1]; Erfan Ghasemi, PhD[1]; Moein Yoosefi, MSc[1]; Negar Rezaei, MD, PhD[1,3]; Yosef Farzi, MSc[1]; Ameneh Kazemi, MSc[1]; Naser Ahmadi, MSc[1]; Maryam Nasserinejad, MSc[1,4]; Nima Fattahi, MD[1]; Shahabeddin Rezaei, MSc[1,5]; Elham Abdolhamidi, MSc[1]; Elmira Foroutan Mehr, MSc[1]; Rosa Haghshenas, BSc[1]; Nazila Rezaei, MD[1]; Jaleh Abdi, MD, PhD[6]; Alireza Moghisi, MD, MPH, PhD[7]; Alireza Mahdavihezaveh, MD, MPH[7]; Ali Akbari Sari, MD, MPH, PhD[6,8]; Alireza Raeisi, MD[7,9]; Hamidreza Jamshidi, PharmD, PhD[10]; Bagher Larijani, MD[3]; Farshad Farzadfar, MD, MPH, MHS, DSc[1,3*]

[1]Non-Communicable Diseases Research Center, Endocrinology and Metabolism Population Sciences Institute, Tehran University of Medical Sciences, Tehran, Iran
[2]Development of Research and Technology Center, Deputy of Research and Technology, Ministry of Health and Medical Education, Tehran, Iran
[3]Endocrinology and Metabolism Research Center, Endocrinology and Metabolism Clinical Sciences Institute, Tehran University of Medical Sciences, Tehran, Iran
[4]Department of Biostatistics, Faculty of Paramedical Sciences, Shahid Beheshti University of Medical Sciences, Tehran, Iran
[5]Ohio State University, Columbus, OH, USA
[6]National Institute for Health Research, Tehran University of Medical Sciences, Tehran, Iran
[7]Deputy of Health, Ministry of Health and Medical Education, Tehran, Iran
[8]Department of Health Management and Economics, School of Public Health, Tehran University of Medical Sciences, Tehran, Iran
[9]School of Medicine, Shiraz University of Medical Sciences, Shiraz, Iran
[10]Research Institute for Endocrine Sciences, School of Medicine, Department of Pharmacology, Shahid Beheshti University of Medical Sciences, Tehran, Iran

Supplementary File 2. The details of the weighting procedure of the cleaned data of Iran STEPS survey 2021.

The weighting procedure after cleaning the gathered data was done in four stages in this survey as follows:

1. Weighting for overall non-response:

The weighting procedure was done in this stage to resolve the problem of individuals who refused to participate in the survey when substituting someone else was not possible because it would make the sex and age structure of the included population heterogenous. In this regard, to cover the non-response problem (after what was done for non-response during the sample size calculation process), weighting was done after the gathered data was cleaned. Regarding the COVID-19 outbreak during the implementation phase of this survey, the total number of samples in each cluster of the sampling survey was 10 before the outbreak and 9 for the phase happening during the pandemic. Calculation of the non-response weighting for the first and second steps of the study, which recruited samples aged ≥18 years old, was conducted using the following equations of (#3) and (#4):

$$(\#3)\ W_{individual\ nonresponse\ questionnare} = \frac{10\ or\ 9}{number\ of\ sample\ in\ cluster}$$

$$(\#4)\ W_{individual\ nonresponse\ anthropometry} = \frac{10\ or\ 9}{number\ of\ sample\ in\ cluster}$$

Regarding the third step of the survey, which recruited individuals aged ≥25 years, since the expected number of participants with this condition was known in the sampling frame of the study, we used the proportion of the individuals aged at least 25 years to estimate the total number of participants in this step and also the non-response weighting according to equation (#5):

$$(\#5)\ W_{individual\ nonresponse\ laboratory} = \frac{People\ over\ the\ age\ of\ 25\ that\ we\ expect\ to\ be\ included\ in\ the\ study.}{number\ of\ sample\ in\ cluster}$$

2. Weighting for non-response in each step:

This stage was done for weighting the data for the specific non-response for each step of the survey including the questionnaires, anthropometry (physical measurements), and laboratory measurements. In cases where the participants refused to answer a part of the questionnaires, the weighting was done based on equation (#6) to prevent data loss and measurement bias:

$$(\#6)\ W_{questionnaire\ nonresponse} = \frac{number\ of\ sample}{number\ of\ sample\ in\ cluster\ with\ complete\ questionnaire}$$

In cases where the participant refused to take part in physical measurements, weighting was done using equation (#7):

$$(\#7)\ W_{anthropometry\ nonresponse} = \frac{number\ of\ sample}{number\ of\ sample\ in\ cluster\ with\ complete\ anthropometry}$$

For the third step of the survey, weighting was done for those who refused to take part in laboratory measurements and also for the participants aged under 25 years since the data of the third step was absent for them. The weighting for this purpose was done using equation (#8):

$$(\#8)\ W_{laboratory\ test\ nonresponse} = \frac{number\ of\ samples\ eligible\ for\ laboratory\ step}{number\ of\ sample\ in\ cluster\ with\ complete\ laboratory\ test}$$

3. Weighting for samples of each province based on age, sex, and area of residence:

This stage of weighting was done to make the included population in each province representative of that province regardless of individual-specific features like age, sex, and area of residence (urban/rural). This stage of weighting was done using the proportion of the number of the population in a selected combination of the mentioned characteristics to the expected number of individuals in samples of each combination of the same using the equations (#9), (#10), and (#11):

$$(\#9)\ W_{province\ questionnare} = \frac{population_{(province,age,sex,area)}}{sum(W_{\substack{questionnaire\ nonresponse\\questionnare}} * W_{individual\ nonresponse})_{(province,age,sex,area)}}$$

$$(\#10)\ W_{province\ anthropometry} = \frac{population_{(province,age,sex,area)}}{sum(W_{\substack{anthropometry\ nonresponse\\anthropometry}} * W_{individual\ nonresponse})_{(province,age,sex,area)}}$$

$$(\#11)\ W_{province\ laboratory} = \frac{population_{(province,age,sex,area)}}{sum(W_{\substack{laboratory\ nonresponse\\laboratory}} * W_{individual\ nonresponse})_{(province,age,sex,area)}}$$

Considering the possible regional differences in age and sex combinations in each province with the population, a further round of adjustments was done using the equation (#12):

$$(\#12)\ W_{adjust} = \frac{\text{Percentage of sample by age,sex,region,and province}}{\text{Percentage of population by age,sex,region,and province}}$$

4. Summarizing and incorporating all weightings into data:

All previously mentioned weightings were summarized in the following equations and added to the gathered and cleaned data of each step of the questionnaires (#13), anthropometry (#14), and laboratory measurements (#15):

$$(\#13)\ W_{Questionnaire} = W_{questionnaire\ nonresponse} * W_{\substack{individual\ nonresponse\\questionnare}} * W_{province\ questionnare} * W_{adjust}$$

$$(\#14)\ W_{Anthropometry} = W_{anthropometry\ nonresponse} * W_{\substack{individual\ nonresponse\\anthropometry}} * W_{province\ anthropometry} * W_{adjust}$$

$$(\#15)\ W_{Laboratory} = W_{laboratory\ nonresponse} * W_{\substack{individual\ nonresponse\\laboratory}} * W_{province\ laboratory} * W_{adjust}$$

The final data after the weighting stages underwent analysis for different variables and indices according to the predefined study aims. Also, they will be used for further investigations in future publications.
